# A systematic review of economic evaluations of whole-genome sequencing for the surveillance of bacterial pathogens

**DOI:** 10.1099/mgen.0.000947

**Published:** 2023-02-15

**Authors:** Vivien Price, Lucky Gift Ngwira, Joseph M. Lewis, Kate S. Baker, Sharon J. Peacock, Elita Jauneikaite, Nicholas Feasey

**Affiliations:** ^1^​ University of Liverpool, Liverpool, UK; ^2^​ Malawi-Liverpool-Wellcome Programme, Blantyre, Malawi; ^3^​ Liverpool School of Tropical Medicine, Liverpool, UK; ^4^​ University of Cambridge, Cambridge, UK; ^5^​ Imperial College London, London, UK

**Keywords:** antimicrobial resistance, economic evaluation, foodborne pathogens, infection prevention and control, systematic review, whole-genome sequencing

## Abstract

Whole-genome sequencing (WGS) has unparalleled ability to distinguish between bacteria, with many public health applications. The generation and analysis of WGS data require significant financial investment. We describe a systematic review summarizing economic analyses of genomic surveillance of bacterial pathogens, reviewing the evidence for economic viability. The protocol was registered on PROSPERO (CRD42021289030). Six databases were searched on 8 November 2021 using terms related to ‘WGS’, ‘population surveillance’ and ‘economic analysis’. Quality was assessed with the Drummond–Jefferson checklist. Following data extraction, a narrative synthesis approach was taken. Six hundred and eighty-one articles were identified, of which 49 proceeded to full-text screening, with 9 selected for inclusion. All had been published since 2019. Heterogeneity was high. Five studies assessed WGS for hospital surveillance and four analysed foodborne pathogens. Four were cost–benefit analyses, one was a cost–utility analysis, one was a cost-effectiveness analysis, one was a combined cost-effectiveness and cost–utility analysis, one combined cost-effectiveness and cost–benefit analyses and one was a partial analysis. All studies supported the use of WGS as a surveillance tool on economic grounds. The available evidence supports the use of WGS for pathogen surveillance but is limited by marked heterogeneity. Further work should include analysis relevant to low- and middle-income countries and should use real-world effectiveness data.

## Data Summary

All articles reviewed in this paper are publicly available, and the detailed search strategy is given in the Table S1 (available in the online version of this article).

**Impact Statement**

The coronavirus disease 2019 (COVID-19) pandemic demonstrated the feasibility and value of large-scale WGS surveillance of severe acute respiratory syndrome coronavirus 2 (SARS CoV-2) to rapidly guide control measures. The upscaling of WGS infrastructure and analytical expertise presents an exciting opportunity to apply WGS-based surveillance to the antimicrobial resistance (AMR) crisis, however the cost-effectiveness of employing WGS for this purpose is underexplored.

Our systematic review identified nine economic analyses of WGS for surveillance of bacterial pathogens relating to use in food safety and in hospital infection prevention and control. All of these studies supported the use of WGS as a surveillance tool on economic grounds. They also emphasized the incremental benefit from earlier generation of WGS-based surveillance data to facilitate the timely implementation of – and thus maximize the benefit from – control strategies informed by the resulting data.

Our findings emphasize the value of WGS-based surveillance of bacterial pathogens, and lend support to the economic case for implementation of programmes aimed at upscaling sequencing capacity for surveillance of AMR.

## Introduction

Whole-genome sequencing (WGS) offers unparalleled insights into the evolutionary history and phylogenetic relationships of pathogens, detection and characterization of resistance genes and other characteristics. The level of discrimination it offers has the potential to revolutionize the surveillance of infectious diseases. For bacterial pathogens, it can identify transmission links, describe outbreaks separated in space and time, exclude outbreaks and provide an understanding of antibiotic resistance in exquisite detail [1]. While such information is unquestionably scientifically valuable, its widescale deployment in clinical diagnostics or for national and international surveillance systems has been constrained in part by cost, lack of necessary equipment, infrastructure, standardization of methods and expertise [2]. The coronavirus disease 2019 (COVID-19) pandemic necessitated massive upscaling in laboratory and bioinformatic expertise and capacity, embedding WGS surveillance into routine practice, demonstrating both the utility and feasibility of large-scale WGS [3], and highlighting the potential for application to other pathogens. The antimicrobial resistance (AMR) crisis is an ever-growing global health concern, with an estimated 4.95 million associated deaths in 2019 [4]. However, the economic realities of large-scale surveillance for AMR remain poorly explored.

WGS for the surveillance of bacterial pathogens has increasingly been applied in a variety of settings, including for public health [5], food safety [6] and hospital infection prevention and control [7]. Surveillance may be targeted at specific pathogens of interest (e.g. the foodborne pathogen *Salmonella enterica *), pathogens with specific resistance profiles (e.g. healthcare-associated multidrug-resistant Enterobacterales) or those implicated in an outbreak. With a few exceptions, WGS surveillance is more commonly employed as a second-line investigation, or to a subset of isolates in the form of sentinel sampling, rather than a sequence-first comprehensive screening system [5].

Another barrier to its implementation is that analyses of economic impact are limited and heterogeneous, and the economic advantage to WGS surveillance is unproven [8]. Economic evaluation is an umbrella term for analyses that consider both the costs and consequences (or benefits) of an intervention and a comparator, although partial evaluations may consider only components thereof (e.g. a costing study, which considers costs but not consequences) [9]. The type of analysis depends on whether it is deemed possible or appropriate to monetize all consequences considered, known as cost–benefit analysis, or whether some parameters cannot be monetized. Non-monetized parameters could be considered in natural units (e.g. blood pressure measurement or progression-free survival) known as cost-effectiveness analysis, or in a standardized composite score such as a quality adjusted life years (QALYs), known as cost-utility analysis [10].

This review aims to comprehensively summarize and review available evidence relating to the economic implications of the use of WGS in the surveillance of bacterial pathogens, following a systematic methodology and reporting framework. Of particular interest was the potential application to antimicrobial-resistant pathogens.

## Methods

### Development of search strategy and identification of relevant articles

The PROSPERO International Prospective Database of Systematic Reviews [11] was searched to identify any in-progress review(s) examining the same topic. The research question and search strategy were proposed and refined in discussion with experts in the field (S.P., N.F., J.L.). The draft protocol was reviewed by two experts in the field of WGS and AMR surveillance (N.F., S.P.) prior to registration on PROSPERO (registration number CRD42021289030). There was no specific funding for this review.

To search for available evidence on the economic evaluation of WGS for the surveillance of bacterial pathogens, six databases [Pubmed, Scopus, EconLit, Cochrane Library, NHS Economic Evaluation Database (NHSEED) and BioRxiv/MedRxiv] were searched on 8 November 2021. Reference lists and articles suggested by experts in the field were also screened for inclusion. Search terms were adapted to the requirements of the database being used (see Table S1). Terms included: ‘WGS’, ‘population surveillance’ and ‘economic analysis’.

Inclusion criteria were: published manuscripts or pre-print literature in English, available in full text between 1 October 1991 and 1 October 2021 with any form of full or partial economic evaluation of WGS for surveillance of one or more bacterial genera and/or species of World Health Organization (WHO)-defined priority pathogens for research and development of new antibiotics (Table S2) [12]. Studies were included whether or not the threshold of drug resistance was met because the cost of sequencing a bacterial genome does not differ with presence or absence of resistance genes, hence the rationale for this criterion. The date range was chosen to span 20 years and encapsulate all relevant studies, as the first complete bacterial genome was published in 1995 [13]. Duplicate studies, those that did not report an economic analysis and those that did not include surveillance for at least one of the priority pathogen species were excluded, due to our focus on antimicrobial resistance. Reviews and other forms of literature not representing primary analyses were not included in the review, although these were considered for background context. Titles and/or abstracts were screened against inclusion criteria by one reviewer (V.P.). Articles selected for full-text review were exported to Rayyan [14] and were assessed against inclusion criteria inclusion by two reviewers working independently (V.P. and L.N.), with disagreements resolved through discussion.

### Data extraction and quality assessment

Data were extracted from each included study by one reviewer (V.P.) into an Microsoft Excel spreadsheet (Redmond, WA, USA), and checked by another (L.N.). Study characteristics [publication year, year of data collection, economic analysis type, country setting, viewpoint, target organism(s), surveillance application, reporting currency, comparator, WGS post per isolate, comparator cost per isolate], methodological details and outcome data were extracted: estimated or actual impact of WGS on burden of illness (accepting any study definition of burden of illness, i.e. cases or deaths averted); the costs and cost savings of WGS programmes; and the results of any break-even analysis. Given the heterogeneity of studies and reporting, a pilot process was used to refine other data extracted, focusing on the methodology as well as the results of trials. The Drummond–Jefferson checklist, developed to improve the clarity of reporting for economic analyses of healthcare interventions [15], was used as an objective measure of quality. The checklist was completed for each study by two reviewers (V.P., L.N.) independently, with disagreements resolved by consensus.

### Data synthesis and reporting

The heterogeneity of economic analysis types, geopolitical contexts, surveillance scales and time points and the limited number of manuscripts precluded formal meta-analysis, so a narrative approach was taken to the synthesis of the methodology and results of the included studies following the recommendations of the Synthesis Without Meta-analysis (SWiM) reporting guideline [16]. Completed PRISMA and SWiM checklists are included in Tables S5–7 [17]. Not all of the extracted outcomes were investigated by all of the studies, so results are presented in a table allowing visualization of heterogeneity. Costs and cost savings are reported in 2020 United States Dollars to enable comparisons, with costs in the currency and year of reporting also shown throughout. Conversions used the average annual exchange rate for the year of reporting according to the Bank of England database [18], and adjusted for inflation to 2020 values using the United States Gross Domestic Product Deflator from The World Bank [19].

In lieu of meta-analysis, a vote count on direction of effect is included. Studies were judged to favour WGS over the comparator where (a) benefits outweighed costs in a cost–benefit analysis, (b) dominance was established in cost-effectiveness analysis and (c) author judgement of realistic case numbers averted in break-even analysis. Costs of illness are presented but not aggregated because frequency of infections due to different pathogens were not reported in all studies.

Heterogeneity is explored through presentation of a method and results table comparing the differing approaches of different studies and the diversity in reporting outcomes. As this review incorporates a variety of forms of economic analysis across a small number of studies, they are not prioritized and are reported in reverse chronological and alphabetical order wherever they appear.

## Results

### Results of search

**Fig. 1.**
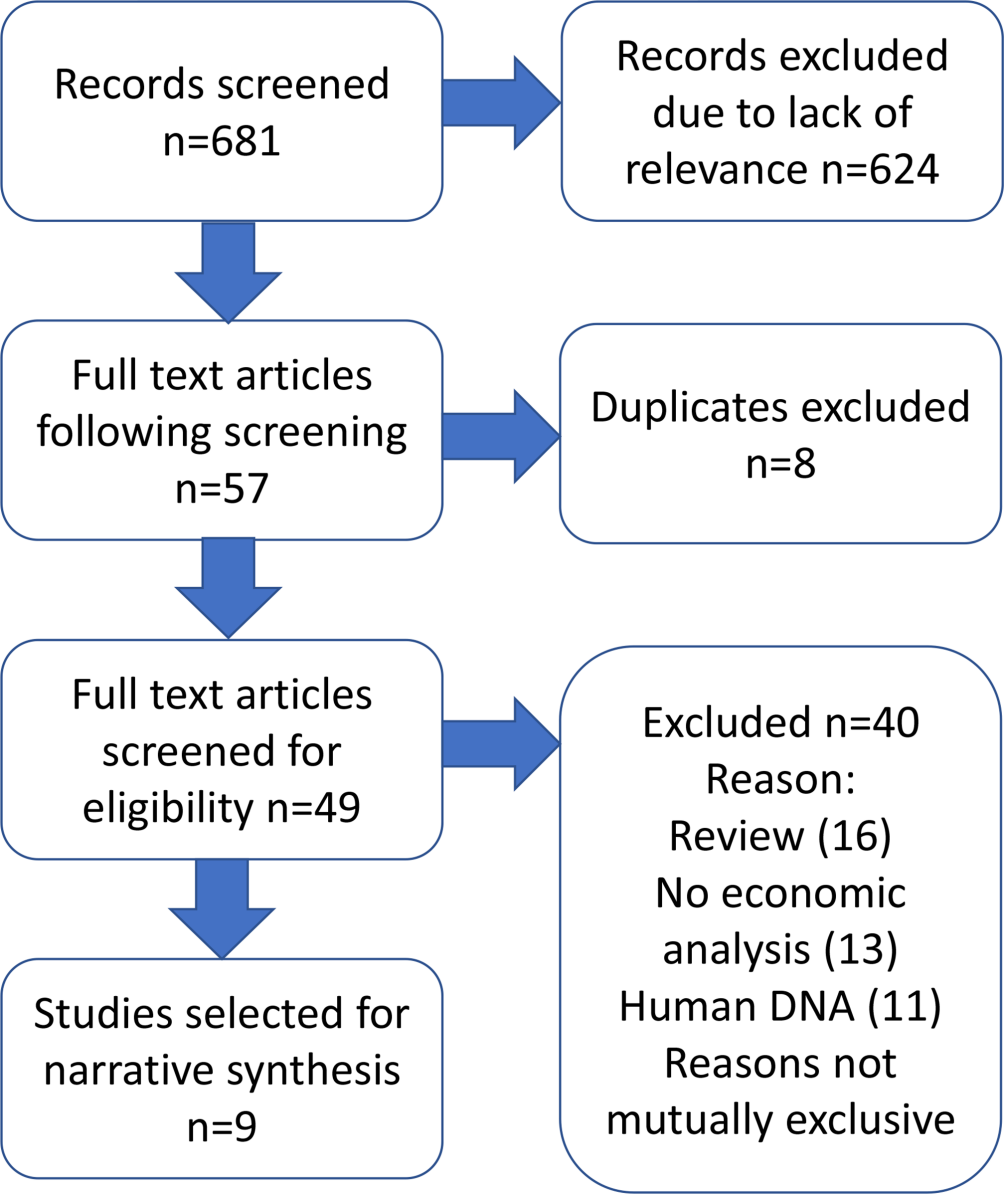
PRISMA flow diagram of included studies. Further detail on excluded studies available in Table S4.

Six hundred and eighty-one studies were generated by the search strategy (Fig. 1, Table S3). Following title and abstract screening, 57 were identified for full-text assessment, of which 8 were duplicates and 49 proceeded to full-text screening. Screening of reference lists from included articles yielded one further article meeting the inclusion criteria. Nine studies were selected for inclusion in this review, with the remainder being excluded for the following reasons: did not include economic analysis (*n*=22); evaluation of organisms other than bacteria (*n*=21); review article not presenting primary analysis (*n*=21) (Fig. 1). In some cases, more than one exclusion criterion applied. A list of excluded studies is included in Table S4.

### Characteristics of included studies

The characteristics of the included studies are summarized in Table 1. Of the nine included studies six [20–25] were published in 2021, with the remaining three [26, 27] published in 2020 and 2019 [28]. The report by Alleweldt *et al*. includes case report analysis of eight reference laboratories, of which the five conducting foodborne bacterial pathogen surveillance were considered, while the remaining three were not, as they reported on viruses and so were not relevant for bacterial AMR [21]. Five studies analysed WGS surveillance for hospital Infection Prevention and Control (IPC), and four studies examined the role of WGS surveillance for foodborne pathogens. All studies reporting on foodborne pathogens considered salmonellosis specifically, with other foodborne pathogens also considered by Alleweldt *et al*. [21] and Brown *et al*. [22]. Of the studies reporting on IPC, those by Gordon *et al*. [25] and Kumar *et al*. [29] reported on WGS surveillance of a variety of pathogens of concern within a hospital, while the remaining studies focused on specific pathogens of interest: methicillin-resistant *Staphylococcus aureus* (MRSA) [26], carbapenem-resistant *Acinetobacter baumannii* [23] and carbapenemase-producing *Escherichia coli [27]*.

**Table 1.** Characteristics of included studies Income level defined according to the World Bank. Currency reported in 2020 USD (and in the currency of primary report)

	Alleweldt *et al*. 2021	Brown *et al*. 2021	Elliot *et al*. 2021	Ford *et al*. 2021	Gordon *et al*. 2021	Kumar *et al*. 2021	Dymond *et al*. 2020	Lee *et al*. 2020	Jain *et al*. 2019
**Data from**	2017 +/−	1999–2019	2016–2018	2018	2021	2011–2016	2012–2013	2017	2000–2015
**Economic analysis type**	Cost–benefit	Cost–benefit	Cost-effectiveness, cost–utility	Costing study	Cost–benefit, cost-effectiveness	Cost-effectiveness	Cost–utility	Cost–benefit	Cost–benefit
**Country of interest**	Various: Argentina, Canada, Italy, UK, USA	USA	Australia	Australia	Australia	USA	UK	Australia	Canada
**Income level of country of interest**	Upper middle income (Argentina), high (others)	High	High	High	High	High	High	High	High
**Viewpoint**	National surveillance	National surveillance	Hospital	National surveillance	Local government	Hospital	Hospital	Hospital	National surveillance
**Target organism**	Foodborne pathogens including *E. coli *, *Listeria *, *Salmonella *, *Shigella * (study also included influenza, not considered in this review)	* E. coli *, *Listeria *, *Salmonella *	Carbapenem-resistant *Acinetobacter baumannii *	* Salmonella enterica *	MRSA, ESBL *E. coli *, VRE, ESBL *K. pneumoniae *, CPE, CRAB	* Acinetobacter baumannii *, *Clostridium difficile *, *Klebsiella pneumoniae *, *Pseudomonas putida *	Methicillin-resistant *Staphylococcus aureus *	MDR *E. coli *	* Salmonella* (non-typhoidal)
**Surveillance application**	Foodborne pathogens	Foodborne pathogens	Hospital infection prevention and control	Foodborne pathogens	Hospital infection prevention and control	Hospital infection prevention and control	Hospital infection prevention and control	Hospital infection prevention and control	Foodborne pathogens
**Reporting currency (year**)	EUR (assumed 2017)	USD (2019)	Australian dollars (2020)	USD (2018)	Australian dollars (base year 2020, costs inflated to 2019)	USD (2018)	GBP (2017/18)	Australian dollars (2018)	Canadian dollars (2015)
**Comparator**	Various, including: serotyping, PFGE, PCR, PCR typing, MLVA, MLST, biochemical analysis, and others	PFGE	Delayed use of WGS+environmental metagenomics	Culture and serotyping, culture and MLVA (study also considered PCR, not considered in this review)	Routine microbiology	Standard infection prevention and control practices, including reactionary WGS	Standard infection prevention and control practices	No WGS, delayed WGS	PFGE
**WGS cost/isolate USD (amount in reporting currency**)	Argentina: 183.90 (154.49) Canada: 256.36 (215.36) Italy: 470.37 (395.14) UK: 148.31 (124.59) USA: 183.92 (154.51)	Not reported	103.27 (150) WGS 244.42 (355) metagenomics	85.67 (83.15)	307.36 (437)	72.13 (70)	134.29 (100)	272.86 (354.70)	115.12 (135.6)*
**Comparator cost/isolate USD (amount in reporting currency**)	*Argentina: 55.48 (46.61)* *Canada: 112.24 (94.29)* *Italy: 109.36 (91.87)* *UK: 77.92 (65.46)* *USA: 96.61 (81.16)*	Not reported	na	Culture 41.85 (40.62) Serotyping 43.66 (42.37) MLVA 54.26 52.66	57.67 (82)	Not specified	Positive 11.00 (8.19) Negative 6.43(4.79)	60.95 (79.23)	219.45 (258.49)*

*Considered cost equal for the purposes of analysis; figures quoted are given in the discussion as an expert opinion on consumable costs.

CPE, carbapenemase-producing Enterobacterales; CRAB, carbapenem-resistant *Acinetobacter baumannii *; ESBL, extended-spectrum beta-lactamase; EUR, euro; GBP, pound sterling; MDR, multidrug-resistant; MLST, multilocus sequence typing; MLVA, multiple locus variable-number tandem repeat analysis; MRSA, methicillin-resistant *Staphylococcus aureus *; PCR, polymerase chain reaction; PFGE, pulsed-field gel electrophoresis; UK, United Kingdom; USA, United States of America; USD, US dollars; VRE, vancomycin-resistant enterococci; WGS, whole-genome sequencin.

Four studies were cost–benefit analyses, one was a cost–utility analysis, one was a cost-effectiveness analysis, one combined cost-effectiveness and cost–utility analyses, one combined cost-effectiveness and cost–benefit analyses and one was a partial analysis (Table 1). Studies were based on data from high-income countries, with the exception in Alleweldt *et al*. which included a case study from Argentina [21]; currently classed as an upper middle-income nation [30].

Where stated, the most common comparator for the laboratory bacterial typing analysis component was pulsed-field gel electrophoresis (PFGE), used by three of the four laboratories looking at foodborne pathogens. In hospital studies, the comparator was deemed to be standard IPC practice, a narrative term incorporating epidemiological linkage, and in some cases reactionary typing.

### Results of economic analyses: non-monetized

The monetized and non-monetized economic analysis outcomes are summarized in Table 2. For the five studies that considered WGS surveillance in hospitals, all reported on either cases (colonization and/or infection) and/or deaths averted. In the three studies estimating deaths averted due to WGS in an individual hospital or hospital trust, estimates ranged from two (MRSA deaths averted) [26] to six (MDR *E. coli *, increased virulence scenario) [27]. Gordon *et al*. estimated 650 deaths averted/year across Queensland, Australia with WGS surveillance [25]. Both Dymond *et al*. [26] and Elliott *et al*. [23] reported a cost–utility analysis including a standardized metric of illness averted in QALYs. Dymond *et al*. estimated a relatively modest 14.28 QALY increment using WGS compared to standard care for MRSA [26], while Elliott *et al*. reported a 59 QALY increment with prior WGS for carbapenem-resistant *Acinetobacter baumannii* (CRAB) [23]. The only other study using QALY as a metric was Jain *et al*.; however, as they only reported QALY and DALY information for baseline data and not for WGS modelling, no conclusions can be drawn [28].

**Table 2.** Monetized and non-monetized outcomes

Study	Impacts on illness	Costs and cost savings	Break-even point	Observations
Alleweldt *et al*, 2021	Actual or estimated cases averted are not collated in this study, although the Supplementary Material includes case reports of outbreaks and effects on burden of disease reported by the participating laboratories	In the break-even analysis, costs per case of salmonellosis ranged from $14 072–15 743) (EUR 11 821–13 225)	Between 1.2 and 82.3 cases (0.2–1.1 %) of salmonellosis, or a single salmonellosis-related death, would need to be prevented to break even with respect to the additional costs of WGS	Study is the most thorough from laboratory costs perspective There was an inverse relationship between batch size and total per-sample costs, whereby larger batches decreased per-sample costs (economy of scale) Early detection of an outbreak with WGS depends on deployment early in the analytical process – if WGS is only performed once the outbreak is confirmed by conventional means, it is likely to be less useful
Brown *et al*, 2021	The most complete model estimates a reduction in observed illnesses of 6.09 for each 1000 isolates added to the NCBI library, while an increase in outbreaks of 0.01 is predicted, with a reduction in illnesses per outbreak of −1.07. Estimates by 2019: reduction in *Listeria* by 210, *E. coli* 5592 and *Salmonella* 19 792 (total 25 595, 95 % CI 9619–43 589)	Total averted by 2019 = $503 million (496.98 million) (95 % CI £190.91–846.03 million), of which $152.79 million (150.96 million) from *Listeria *, $1.93 million (1.91 million) from *E. coli* and $3.37 million (3.33 million) from *Salmonella * Cost of running programme 21.56 million (21.3 million) per year. Estimated net benefits in 2019 of $126.51 million (USD 125 million)	Source-tracking programme likely broke even in second year, in which estimated cases averted were 31 *Listeria *, 185 *E. coli *, 574 *Salmonella *, with respective estimated averted costs of $51.57 million (50.95 million), $1.7 million (1.68 million) and $2.86 million (2.83 million) (total $56.13 million (55.46 million) ($21.04—96.04 million, 20.79–94.89 million)	Figures include multiplier for under-reporting and under-diagnosis Surveillance initially targeted *Listeria *, which accounts for greater cost savings despite lower case numbers due to higher mortality Coupling benefits to pathogen–ood vehicle pairs would help to understand how WGS can be targeted
Elliott *et al*, 2021	Prior use of WGS and delayed use of environmental metagenomics (scenario 2) resulted in 14 fewer patients with CRAB and 59 additional QALYs Prior use of both WGS and environmental metagenomics (scenario 3) resulted in 18 fewer patients with CRAB and 74 additional QALYs	Prior use of WGS and delayed use of environmental metagenomics (scenario 2) resulted in $51 706 (A$75 099) cost savings Prior use of both WGS and environmental metagenomics (scenario 3) resulted in $64 598 (A$93 822) cost savings	Not calculated	Specific focus on environmental contamination WGS was a small fraction of the total hospital costs (~2%), and changing the cost of sequencing in the sensitivity analysis did not change the outcome
Ford *et al*, 2021	While the modelled outbreak scenarios rely on averted cases for cost-savings calculated, these are not reported	Cost per case (infection) was $1131 (1098) In modelled outbreaks, no cost saving with WGS was predicted for a point source outbreak (culture and serotyping $37 761 (36 648), WGS $39 000 (37 851), in a prolonged outbreak with no peak WGS was predicted to result in savings of $44 114 (42 814), $108 247 (105 057) or $151 165 (146 711) and in a prolonged outbreak with late peak savings of $136 769 (132 739), $190 660 (185 042) or $246 100 (238 848) compared to culture and serotyping based on intervention at 30, 60 or 90 days earlier	275 (90 % CI −55–775) or 1.9 % (90 % CI −0.4–5.4 %) of all notified serotyped *Salmonella* cases needed to be prevented for WGS to be cost-equal to serotyping and MLVA	WGS was already cheaper than serotyping and MLVA for *Salmonella* Typhimurium Comparisons with PCR lack relevance due to the inferior acuity of data produced
Gordon *et al*, 2021	WGS surveillance predicted to avert: In first year: 2085 infections, and 34 641 colonizations, and 650 deaths Across 5 years: 9605 infections, 149 669 colonizations and 2880 deaths	WGS surveillance microbiology and sequencing costs estimated $13.01 million (A$18.5 million) more than standard of care; offset by savings in delivery of care Total cost savings $21.73 million (A$30.9 million) in year 1, dropping to $15.54 million (A$22.1 million) by year 5 Cost saved for each avoided infection was $4865 (A$6917), and for each colonization was $334 (A$475) in year 1	Not calculated	Projected cost savings are dependent on successful action by infection control teams Sensitivity analysis most sensitive to variation in estimates of preventable infections Consideration of societal cost savings and other benefits of WGS data (e.g. industry and research benefits) Sequencing costs high, but authors note in discussion that streamlining workflows could reduce cost per isolate to A$150
Kumar *et al*, 2021	Across 11 outbreaks, WGS predicted to avert 41 transmissions three deaths	Cost of running WGS programme $490 380 (475 930 /year) Estimated savings of $502 555 (487 747) in treatment costs (based on 11 900/transmission averted) Net savings of $9 348 (9073) (discounted) WGS surveillance less costly and more effective than standard practice in base case; in probabilistic sensitivity analysis WGS was cost saving and more effective in 49% of the simulations	Not calculated	Reportedly results most sensitive to cost of performing WGS in sensitivity analysis; as assumed cost/sample was $72 (70), this may have an impact on applicability in settings where WGS is more costly 80 % chance that WGS would be cost-effective if willingness to pay exceeded 2473 (2400) per transmission averted
Dymond *et al*, 2020	Across 1 year (65 000 patients), WGS predicted to avert 249 asymptomatic colonization, 41 infections and 2 MRSA related deaths Absolute increase in QALYs with WGS 14.28	Cost of WGS surveillance $3.17 million (£2.36 million) compared to standard practice $4.15 million (£3.09 million), resulting in absolute saving of $977 997 (£728 297) with WGS Majority of cost savings due to MRSA-related treatment costs $2.7 million (£1.97 million) WGS dominant to standard practice	Sequencing predicted to be cost-effective as long as effectiveness >30 %	Results most sensitive to changes in probability of MRSA acquisition, which was an assumed parameter
Lee *et al*, 2020	With no WGS, 352 patients colonized and 902 bed closures; with delayed WGS 197 colonized patients and 419 bed closures; with early WGS 3 patients colonized and 11 bed closures. In the environmental contamination scenario, 234 patients were predicted to become colonized without use of WGS, compared to two with early use of WGS. In the increased virulence scenario, 256 patients were predicted to become colonized, 41 to develop infection and six to die without use of WGS; with early WGS predictions were 3 colonized and one infection (no deaths).	Cost savings with delayed WGS were $236 001 (A$306 785) and with early WGS were $539 697 (A$701 547), compared to not using WGS Environmental contamination scenario: savings of $451 299 (A$586 659) with early WGS compared to no WGS Increased virulence scenario: savings of $621 745 (A$808 227) with early WGS compared to no WGS	Not calculated	Microbiology screening accounted for the most significant single cost area As a single-pathogen, single-hospital study there may be limits to generalizability; however, this may also result in the underestimation of benefits as well as opportunities to take advantage of economies of scale
Jain *et al*, 2019	While the model relies on cases averted, absolute numbers are not reported. DALYS and QALYs are reported for Salmonellosis (529.20 and 289.90), however there is no calculation for estimated effect of WGS	Total costs of WGS predicted to be cheaper than PFGE $167.69 million vs $244.31 million (CAD 197.52 million vs 287.78 million), with net benefits of $76.62 million (CAD 90.25 million) or $55.17 million (CAD 64.98 million) with a reduction in illness cases by 70 or 50%, and net benefits of $4.42 (CAD 5.21 million) if only reported cases taken into account	Not calculated	Model does not account for any direct cost difference between the two technologies compared (equipment, personnel, etc.), and only mentions in the discussion that expert opinion estimates consumable costs to be lower for WGS than PFGE, $115.12 vs $219.45 (CAD 135.6 vs 258.49) DALYs and QALYs calculated for PFGE but not for WGS

For the analyses of foodborne pathogens, detailed data on cases averted were not presented. Instead, these were considered in aggregate, in the form of cost savings by all except Brown *et al*. who reported illnesses averted by pathogen across the 5 years of their programme: *Listeria* 210, *E. coli* 5592 and *Salmonella* 19 792 (total 25 595 95 % CI 9619–43 589) [22].

As most studies report higher laboratory costs with WGS compared to comparators, the benefits derive from the ability of WGS to avert cases of illness. This is achieved through providing actionable data of a higher acuity or in a faster time frame, enabling effective intervention to prevent further infections. These data do not lend themselves to collation, as the epidemiology of the considered infections, the time horizons and the economic impacts of preventing infection differ.

### WGS costs

Estimates relating to the cost of WGS per isolate were reported in eight of the nine studies (summarized in Table 1). Alleweldt *et al*. [21] report these separately for all five included laboratories, while for Jain *et al*. [28] the estimate is only included in the discussion and is for consumable costs alone. The estimates range from $72.13 by Kumar *et al*. (USA) [29] to $470.37 in the Italian case study from Alleweldt *et al*. [21], with a mean of cost of $194.46. Alleweldt *et al*. [21] provide the most detailed breakdown of the costs considered in relation to providing a cost per isolate for WGS, and in addition to equipment, consumables and staffing they also explicitly consider equipment use and batch size as a factor. Considering this more complete methodology, it may be considered that their estimates of per-sample costs, ranging from $148.31–470.37, are more robust than the other included studies. There was no apparent temporal pattern of cost per isolate becoming cheaper over time, likely reflecting the short publication time span, and cost drivers could not be identified.

### Results of economic analyses: monetized

The heterogeneity in the contexts, studies and reporting make comparisons of total costs and costs averted impossible. Net savings at the level of individual hospitals or hospital trusts ranged from 9348 [29] to $977 997 [26]. At the hospital scale, Kumar *et al*. [29] found WGS to be less costly and more effective than standard care in their base case. However, it should be noted that the estimated sequencing costs quoted were the lowest of all studies included at $72.13 per isolate, within which they did not detail whether additional equipment or staffing costs were considered. Dymond *et al*. [26] identified that WGS was more effective and less costly than current practice. This was, however, based on a high efficacy of 90 % case reduction in the base case and >30 % in the sensitivity analysis. Considering these limitations, the results should be reviewed with caution. Elliott *et al*. estimated cost savings of between 51 706 to $64 598 with environmental metagenomics in addition to WGS surveillance in a modelled CRAB outbreak, but did not include a scenario for WGS surveillance alone [23], limiting comparison with the other studies. Net savings were $8.72 million in the first year of screening for six drug-resistant organisms across Queensland, Australia [25], and $539 697 for a modelled outbreak of MRD *E. coli* for a single Australian hospital [27].

In the studies evaluating foodborne pathogens, the cost per case of salmonellosis was estimated in two studies: $1131 in Australia [24] and $14 072–15 743 across a variety of settings reported by Alleweldt *et al*. [21] These studies also included a break-even analysis. Alleweldt *et al*. [21], combining analysis from five laboratories, estimated that between 1.2 and 82.3 cases (CI 0.2–1.1 %) of salmonellosis, or a single salmonellosis-related death, would need to be prevented to break even, while Ford *et al*. estimated that 275 cases (90 % CI −55–775) or 1.9 % (90 % CI −0.4–5.4 %) of disease would need to be prevented [24]. Although Brown *et al*. [22] did not report a specific break-even analysis for *Salmonella *, they did estimate an overall break-even point for all studied pathogens in year 2 of their programme. Furthermore, the authors state that the break-even figures modelled by Alleweldt *et al*. [21] and Ford *et al*. [24] are likely to be achievable within the early stages of WGS surveillance implementation, according to their model of salmonellosis cases averted.

Assessing foodborne pathogen surveillance, Brown *et al*. [22] report on cost–benefit analysis using their actual data since implementation of routine WGS surveillance, while Ford *et al*. [24] and Jain *et al*. [28] used modelled scenarios. In view of the superior power of WGS over traditional epidemiology in the detection of outbreaks [31], we believe that the use of observed actual data from real-world application of WGS is likely to be more reliable than modelled estimates. Brown *et al*. [22] report $503 million in savings by 2019, by which time their WGS surveillance programme had 5 years of maturity. Ford *et al*. [24] modelled different outbreak scenarios, concluding that for point source outbreaks, WGS would be more costly and no more effective, on the basis that intervention is not possible to avert cases in this type of outbreak. In more prolonged outbreaks, savings of between $44 114–246 100 were predicted, dependent on the type of outbreak and the point at which WGS would allow implementation of effective intervention. Jain *et al*.’s model [28] concluded that WGS was less costly than standard practice and therefore only reported in terms of net benefits. However, their model did not account for any increase in costs with WGS and relied on expert opinion, mentioned only in the discussion, that WGS consumables would be less costly than the comparator. The Jain *et al*. model [28] does not account for the non-consumable costs associated with WGS, including technology and human resources, and is not reflective of the findings of the other included studies, where the per-isolate cost of WGS was generally more expensive than comparators.

The methodological approaches of the studies were diverse (summarized in Table 3).

**Table 3.** Methodological details of studies

Study	Analysis type	Methods employed	Details of method and any model used	Key limitations
Alleweldt *et al*. 2021	Cost–benefit analysis of WGS for pathogen surveillance across different public health laboratories in different countries	Case study of eight reference laboratories routinely using WGS, of which the five using WGS for bacterial pathogens are considered here Costs of WGS compared to next-best conventional method calculated Break-even analysis	Costs of WGS and conventional methods considered from laboratory perspective: equipment, consumables, staff and other costs, and included estimated lifespans, rate of use and maintenance Cost of illness included healthcare utilization, productivity loss and premature death (value of statistical life method) Break-even analysis considered salmonellosis	Underdiagnosis/underreporting multiplier not used, therefore total cost of illness likely underestimated, and break-even point likely overestimated
Brown *et al*. 2021	Cost–benefit analysis of WGS for foodborne illness source tracking in the USA	Theoretical model using social welfare maximization framework Tested in an empirical model using data from FDA and NCBI databases	Theoretical model included: net value of food production minus the total burden of foodborne illness associated with food production, minus the implementation costs of the programme Empirical models included: pathogen, year, average illnesses per outbreak and the no. of sequences for the pathogen in NCBI in given year, food vehicle implicated, indicators to establish effects of Food Safety Modernization Act in different years Cost–benefit model: reduction in illness with increases in WGS isolates in NCBI, underreporting/underdiagnosis multiplier and estimated burden of illness related to each pathogen	Model may not account for complete societal costs of foodborne illness cases
Elliott *et al*. 2021	Cost-effectiveness/cost–utility analysis of WGS and environmental metagenomics for surveillance and management of CRAB in an Australian hospital	A hybrid agent-based and discrete-event model looking at use of WGS and environmental metagenomics in three scenarios modelled using observed outbreak data Healthcare costs and health utility considered	Used AnyLogic (AnyLogic, Chicago, IL, USA) to model the burns unit over 32 months Scenario 1 used actual time point – WGS of CRAB isolates commencing shortly after the outbreak was detected, and environmental metagenomics being introduced more than 1 year later. Scenario 2 assumed WGS use prior to the start of the outbreak and delayed introduction of environmental metagenomics, and scenario 3 assumed prior WGS and metagenomics use	Model based on a single outbreak, single pathogen and single unit, limiting generalizability
Ford *et al*. 2021	Cost analysis of WGS for public health surveillance of non-typhoidal *Salmonella enterica* in Australia	Costs per case calculated for notified cases Cost per sample calculated based on billing by five reference laboratories Break-even analysis for WGS vs serotyping and MLVA Costs modelled in three simulated outbreak scenarios	Cost per case included direct and indirect healthcare costs, productivity lost, premature mortality Modelled scenarios: point source outbreak, prolonged outbreak without peak, prolonged outbreak with late peak. In lieu of an effectiveness estimate, intervention at 30, 60 or 90 days earlier on the epidemiological curve was modelled	Effectiveness estimate not used – unclear how intervention points in modelled outbreak scenarios may apply in real outbreaks Underreporting/underdiagnosis multiplier not used, therefore societal costs likely underestimated
Gordon *et al*. 2021	Cost–benefit/cost-effectiveness of surveillance for six common multidrug-resistant bacteria across hospitals in Queensland, Australia	National data on HAI (9.9%) subdivided by organism and site used to estimate colonizations, infections and deaths in Queensland (16 hospitals) Sequencing data derived from WGS surveillance project performed for research purposes locally (1783 isolates). An SNP threshold <5/Mb used to define a cluster, of which 2–18 clusters identified per pathogen involving 5–13 patients Five-year budget impact analysis – costs for provision of care	Model accounted for changes in resistance rates over the time period Sensitivity analysis performed	Assumption that transmission is successfully broken when WGS data are acted upon, with turnaround time of 7 days for data Sequencing set-up costs not included
Kumar *et al*. 2021	Cost-effectiveness study of WGS for infection prevention and control in a US hospital	Transmission network for 11 outbreaks (1 hospital) involving 89 patients built Transmissions averted by WGS (compared to standard of care) estimated from model Deaths averted estimated Change in cost calculated	Estimates of transmissions averted based on effectiveness of intervening against the suspected transmission route, time from transmission to positive culture and time from time to obtain and act on WGS results Deaths averted calculated by attributable mortality to infections and different anatomical sites Costs included sequencing, staffing for the IPC team and treatment of infections	Deaths averted not considered in economic terms (value of statistical life) Sequencing cost per isolate considered $72.13 (70), which is the lowest in this review Higher acuity of WGS in identifying outbreaks means likely underestimate of benefits due to detection of occult outbreaks/transmission
Dymond *et al*. 2020	Cost–utility study of WGS surveillance of *Staphylococcus aureus* in hospitalized inpatients in the UK	Estimation of MRSA cases averted Costs of treatment of infection or colonization, costs of sequencing and collecting samples were calculated Mortality and QALYs estimated	MRSA case numbers taken from previous prospective cohort study (one laboratory covering three hospitals) Probability of MRSA acquisition assumed 0.5 % and reduction in MRSA due to genome sequencing assumed 90% Cost of WGS $134.29 (£100)/isolate, cost of ITU care and cost of colonization only considered Mortality estimate from Klein *et al*. 2019, QALY decrement 0.35	Probability of MRSA acquisition and the estimated efficacy of WGS are both hypothetical figures, with efficacy of WGS assumed 90%, although addressed to some extent in sensitivity analysis Cost of treating infection assumed intensive care admission, resulting in overestimation. As cost of treatment savings the major driver of cost savings in model, this may have important implications
Lee *et al*. 2020	Cost–benefit analysis of WGS surveillance for MRD *E. coli* in a hospital outbreak in Queensland, Australia	Stochastic hybrid discrete-event, agent-based model using AnyLogic software Evaluating early vs no WGS in different outbreak scenarios (also includes delayed use of WGS): actual outbreak data, simulated outbreak with environmental transmission and outbreak with an increased virulence pathogen	Considered healthcare costs including environmental cleaning, bed closure costs and outbreak team costs	Costs for treating infection (and death) only included in scenario of increased virulence The authors note that the outbreak is modelled on an actual OXA-181 outbreak that did not cause clinical infection in any patients
Jain *et al*. 2019	Cost–benefit analysis of WGS for public health surveillance of non-typhoidal *Salmonella enterica* from specific food vehicles (fresh produce, poultry and eggs) in Canada	Salmonellosis cases attributable to fresh produce, poultry and eggs estimated Cost of illness estimated for PFGE and WGS Three scenarios modelled DALYs and QALYs calculated for PFGE but NOT for WGS	Notified cases, underdiagnosis/underreporting multiplier, and likely percentage attributable to food vehicles of interest used to estimate case numbers – severity of illness calculated with estimates from Hoffman *et al*. 2012 Cost of illness included direct and indirect costs, premature mortality, costs of surveillance calculations use estimates from Thomas *et al*. 2015, QALYs (derived from Hoffman *et al*. 2012) and DALYs (derived from [5]) Modelled scenarios: two historic outbreaks used to model effect of (1) 70 % reduction in cases, (2) 50 % reduction in cases, (3) net benefits for all notified cases in Canada	Considered food vehicles account for 49 % of salmonellosis cases in Canada Model does not account for any direct cost difference between the two technologies compared (equipment, personnel, etc), and only mentions in the discussion that expert opinion estimates consumable costs to be lower for WGS than PFGE $115.12 vs $219.45 per isolate (135.6 vs 258.49), with $257 098.95 (302 837) in equipment costs DALYs and QALYs calculated for PFGE but not for WGS

### Vote count on direction of effect

In view of the lack of a unifying outcome measure reported by the studies, a vote count on the direction of effect was used to enable comparison. All included studies favoured the use of WGS over comparators. A summary of the results of studies considered in terms of illnesses averted, costs averted and any break-even analysis is presented in Table 2.

### Assessment of quality with Drummond–Jefferson checklist

The complete quality assessment using the Drummond–Jefferson checklist across the three areas of study design, data collection, and analysis and interpretation of results is presented in Table 4. All studies scored highly on criteria relating to the study design and justification. There was a high level of transparency in establishing and reporting currency and price data, providing details of models used and establishing benefits. Establishing and reporting primary outcomes, providing an answer to the study question, and conclusions following from the data were also presented well, and in most cases, limitations were appropriately discussed.

**Table 4.** Quality assessment with Drummond–Jefferson checklist

		Alleweldt *et al*. 2021	Brown *et al*. 2021	Elliott *et al*. 2021	Ford *et al*. 2021	Gordon *et al*. 2021	Kumar *et al*. 2021	Dymond *et al*. 2020	Lee *et al*. 2020	Jain *et al*. 2019
**Study design**										
1	The research question is stated	X	X	X	X	X	X	X	X	X
2	The economic importance of the research question is stated	X	X	X	X	X	X	X	X	X
3	The viewpoint(s) of the analysis are clearly stated and justified	X	X	X	X	X	X	X	X	X
4	The rationale for choosing alternative programmes or interventions compared is stated	X	X	X	X	X	X	X	X	X
5	The alternatives being compared are clearly described	X	X	X	X	X	X	X	X	X
6	The form of economic evaluation used is stated	X	X	X	X	X	X	X	X	X
7	The choice of form of economic evaluation is justified in relation to the questions addressed	X	X	X	X	X	X	X	X	X
**Data collection**										
8	The source(s) of effectiveness estimates used are stated		X	X		X	X	X	X	X
9	Details of the design and results of effectiveness study are given (if based on a single study)			X		X			X	X
10	Details of the methods of synthesis or meta-analysis of estimates are given (if based on a synthesis of a number of effectiveness studies)									
11	The primary outcome measure(s) for the economic evaluation are clearly stated	X	X	X	X	X	X	X	X	X
12	Methods to value benefits are stated	X	X	X	X	X	X	X	X	X
13	Details of the subjects from whom valuations were obtained were given	X		X		X			X	X
14	Productivity changes (if included) are reported separately	X								X
15	The relevance of productivity changes to the study question is discussed									X
16	Quantities of resource use are reported separately from their unit costs	X					X		X	
17	Methods for the estimation of quantities and unit costs are described	X			X		X	X	X	X
18	Currency and price data are reported	X	X	X	X	X	X	X	X	X
19	Details of currency of price adjustments for inflation or currency conversion are given		X	X	X	X		X		
20	Details of any model used are given		X	X	X	X	X	X	X	X
21	The choice of model used and the key parameters on which it is based are justified	X	X	X	X	X	X	X	X	X
**Analysis and interpretation of results**										
22	Time horizon of costs and benefits is stated	X	X				X			
23	The discount rate(s) are stated			X			X			
24	The choice of discount rate(s) is justified			X			X			
25	An explanation is given if costs and benefits are not discounted			X				X		
26	Details of statistical tests and confidence intervals are given for stochastic data		X	X	X	X				
27	The approach to sensitivity analysis is given	X		X		X	X	X	X	
28	The choice of variables for sensitivity analysis is justified	X				X	X	X	X	
29	The ranges over which the variables vary are justified	X		X		X	X	X	X	X
30	Relevant alternatives are compared	X	X	X	X	X	X	X	X	X
31	Incremental analysis is reported	X		X			X	X		
32	Major outcomes are presented in a disaggregated as well as aggregated form	X		X						
33	The answer to the study question is given	X	X	X	X	X	X	X	X	X
34	Conclusions follow from the data reported	X	X	X	X	X	X	X	X	X
35	Conclusions are accompanied by the appropriate caveats	X	X	X	X	X	X	X	X	

Effectiveness estimates were not reported by all studies, and in some cases assumed values were used. Where effectiveness estimates were used, sources were generally not described in detail or critiqued. Quantities of resource use were generally not reported. Productivity changes and their relevance to the study question were generally poorly reported. Most studies did not discuss adjustments for inflation or discounting, although this may in part be accounted for by the short time horizons used. Data were generally not presented in a disaggregated form.

The costing study by Ford *et al*. [24] represents a partial rather than full economic evaluation, which is reflected in the checklist. The checklist is intended to guide a narrative assessment of the quality of studies rather than prescribe cut-off values to judge a study as high, medium, or low quality. Overall, the studies were considered to represent high-quality works, with relatively minor variation in quality between them.

## Discussion

This review found nine economic analyses of WGS for bacterial pathogen surveillance, of which five evaluated hospital surveillance and four evaluated foodborne pathogen surveillance. The available evidence for the potential economic benefit of whole-genome sequencing in AMR pathogen surveillance is heterogenous and of varying completeness, but broadly suggests that WGS can be economically viable from the public health perspective of foodborne illnesses, and at the smaller scale of hospital IPC. We found that costs for a single WGS test ranged from 72.13 to $470.37. Over the short timescale of the included studies (2019–2021) there was no evidence of WGS cost falling over time. In addition, there was no apparent regional variation in WGS cost, although all studies were in high- and upper middle-income nations with good supply chains. These costs are broadly in keeping with the costs per isolate identified by Raven *et al*. who reported prices for commercial sequencing of MRSA ranging between GBP 155–342 (226–498 in 2020 USD) per isolate [32].

Most identified studies demonstrated cost savings due to WGS that were largely attributed to averted cases of infection. For this benefit to be realized maximally, WGS needs to be employed early in the analytical pipeline. Conversely, delay in the use of WGS reduces the benefits, as early detection of outbreaks enables timely implementation of interventions to interrupt transmission. Overall, effectiveness estimates were not always used in analysis, or were assumed values. Future economic analyses of WGS should increasingly be able to use effectiveness measures from actual WGS surveillance programmes, rather than relying on assumed or modelled values.

The five studies that looked specifically at AMR pathogens all focused on hospitals and used historic data to model the impact of WGS. The use of real-life outbreak data inherently underestimates the role of WGS, as it limits analysis to outbreaks detected by standard practice, which is less discriminatory than WGS and cannot take account of the potential of WGS to detect outbreaks that are currently evading detection. Future studies assessing the impact of WGS on hospital IPC should use actual data on the effectiveness of WGS surveillance rather than using historic outbreak data derived by conventional means.

No studies were identified that economically evaluated national or regional WGS-based surveillance specifically for AMR-pathogens, though such surveillance programmes are being implemented worldwide. The surveillance of foodborne pathogens did not focus specifically on resistant pathogens, but the methodology for modelling outbreaks has clear potential to be replicated for AMR. Future studies are needed focusing specifically on the application of WGS surveillance to AMR.

Modelling the impact of adding additional WGS data into the National Center for Biotechnology Information (NCBI) database by Brown *et al*. shows the potential for international benefits from WGS surveillance [22]. These authors refer to this as a ‘global food shield’, taking a One Health perspective that could be highly valuable in source tracking. The addition of isolate data to publicly accessible databases also presents the opportunity to benefit research and industry in ways not measured by the included studies. In this work, the large contribution of averted listeriosis cases to the economic benefits, despite low case numbers, demonstrates the significant bearing that severity of illness and mortality rate have. This is important to consider in determining the pathogens for which WGS surveillance can be most effectively utilized.

Almost all of the settings evaluated were high income, but the burden of both foodborne illness and AMR is higher in low and middle-income countries (LMIC), particularly in regions of Africa [4, 33]. Understanding the costs and benefits of WGS surveillance in LMIC settings could have important implications not only for the health of these populations, but also for global efforts to tackle infectious illness and in particular AMR [34], as highlighted in the WHO Global Genomic Surveillance Strategy [35].

The most detailed analysis of the costs of WGS surveillance was in Alleweldt *et al*. which was the only study to report in detail the costs of equipment, consumables and resources, and equipment use, and to consider returns of scale [21]. As sequencing capacity and expertise increases, the costs of sequencing are likely to fall, reflecting competition between suppliers, increased automation and economies of scale [36]. That a fall in sequencing costs was not seen in this review is likely indicative of the short timescale during which all included studies were published. Overall, studies were of high quality, as assessed by the Drummond–Jefferson checklist criteria.

This review has several limitations. Inclusion and exclusion criteria were intentionally broad as a paucity of relevant literature was predicted. This resulted in marked heterogeneity, limiting the ability to make direct comparisons or aggregate results, precluding meta-analysis. However, in view of the inherent differences in economic relationships across different time points and geopolitical contexts, it is likely that meta-analysis to determine a ‘true effect’ would be inappropriate, even if highly comparable outcomes were reported. For example, in considering foodborne pathogens, a host of factors, including climate, aetiology and healthcare services may account for different outcomes in different regions. The differences in study design and methodology also make comparisons of quality assessment challenging, and this has been highlighted previously [37]. The decision to focus on pathogens within the WHO list of priority pathogens for research and development of new antibiotics means that some important pathogens for which WGS surveillance is utilized were excluded, notably *Mycobacterium tuberculosis* (TB). An analysis of the economic impact of routine WGS with molecular diagnostics for TB in the UK estimated an incremental net benefit of GBP 16.6 million (24.17 million in 2020 USD) over a 10-year horizon [38]. The decision not to include TB in this review was taken in view of the significant biological and genetic differences between mycobacterial species and the bacteria more commonly causing foodborne or hospital-associated infections. While a decision to perform narrative synthesis was made *a priori*, the form of synthesis was determined post-hoc, with potential for resulting inadvertent bias. It is possible that economic analyses of WGS surveillance showing negative implications may be less likely to be published, and we were unable to assess for this potential publication bias in this review.

That all articles in this review were published since 2019 – and most during 2021 – speaks to the growing interest in the economics of using WGS surveillance on a large scale. Evaluating the economics of genomics for AMR surveillance has been identified as a priority area for action by the Surveillance and Epidemiology of Drug Resistant Infections Consortium (SEDRIC) genomics working group [38]. It is likely that the evidence base will continue to grow rapidly over the coming years, as a sequence-first paradigm is adopted across more real-world settings. It will be important to see a diverse range of surveillance uses, pathogens and geopolitical contexts represented, to understand how this powerful resource can best be used to the benefit of public health. At this point in time, the available evidence paints a positive view of the economic feasibility of large-scale WGS surveillance.

## Supplementary Data

Supplementary material 1
