# Supplementary material for: A systematic review of economic evaluations of whole-genome sequencing for the surveillance of bacterial pathogens
Source: Microb Genom. 2023 Feb 15;9(2):mgen000947. doi: 10.1099/mgen.0.000947 (PMC9997737; doi:10.1099/mgen.0.000947)
Supplement: Supplementary material 1 [file mgen-9-947-s001.pdf]

## Supplementary material

**Supplementary methods:**

**S Table 1:**
Exact search terms and qualifiers used for each database.

| Database | Search terms |
|---|---|
| **Pubmed** | (“WGS” OR “whole genome sequencing” OR “NGS” or “next generation sequencing”)<br>AND<br>(“environmental monitoring” OR “population surveillance” OR “sentinel surveillance” OR “public health surveillance” OR “epidemiological monitoring” OR “cross infection” OR “healthcare associated infection” OR “HCAI” OR “disease outbreaks” OR “communicable disease control” OR “epidemics” OR “pandemics” OR “food safety” OR “food microbiology” OR “surveillance” OR “public health” OR “screening”)<br>AND<br>(“cost-benefit analysis” OR cost effectiveness OR “economic evaluation” OR “financial evaluation”)<br>NOT<br>(“Mycobacterium tuberculosis” OR “tuberculosis”) |
| **Scopus** | (wgs OR {whole genome sequencing} OR ngs OR {next generation sequencing} AND {environmental monitoring} OR {population surveillance} OR {sentinel surveillance} OR {public health surveillance} OR {epidemiological monitoring} OR {cross infection} OR {healthcare associated infection} OR hcai OR {disease outbreaks} OR {communicable disease control} OR epidemics OR pandemics OR {food safety} OR {food microbiology} OR surveillance OR {public health} OR screening AND {cost-benefit analysis} OR {cost effectiveness} OR {economic evaluation} OR {financial evaluation} AND NOT {Mycobacterium tuberculosis} OR tuberculosis ) |
| **EconLit** | WGS or “whole genome sequencing” |
| **NHSEED** | WGS or “whole genome sequencing” or “sequencing” |
| **Cochrane** | WGS or “whole genome sequencing” |
| **Bio/MedRxiv** | "(WGS OR whole genome sequencing) AND (economic evaluation OR financial evaluation) AND (AMR or "anti* resistance")" |

**S Table 2:**

Bacteria listed on the World Health Organisation (WHO) list of priority pathogens for the research and development of new antibiotics.

| **WHO Priority Pathogens** |
|---|
| *Acinetobacter baumannii* (carbapenem resistant) |
| *Pseudomonas aeruginosa* (carbapenem resistant) |
| *Enterobacteriaceae* (carbapenem-resistant, ESBL-producing) |
| *Enterococcus faecium* (vancomycin-resistant) |
| *Staphylococcus aureus* (methicillin resistant, vancomycin intermediate or resistant*)* |
| *Helicobacter pylori* (clarithromycin resistant) |
| *Campylobacter spp.* (fluoroquinolone resistant) |
| *Salmonellae* (fluoroquinolone resistant) |
| *Neisseria gonorrhoeae* (cephalosporin resistant, fluoroquinolone resistant) |
| *Streptococcus pneumoniae* (penicillin non-susceptible) |
| *Haemophilus influenzae* (ampicillin resistant) |
| *Shigella spp.* (fluoroquinolone resistant) |

**Supplementary results:**

**S Table 3:**

Preliminary search results and number of studies proceeding to full-text screening

| **Database** | **Search results (n=)** | **Selected for full-text screening (n=)** |
|---|---|---|
| Pubmed | 100 | 24 |
| Scopus | 355 | 25 |
| EconLit | 10 | 2 |
| NHS EED | 19 | 0 |
| Cochrane | 0 | 0 |
| Bio/MedRxiv | 191 | 0 |
| Expert recommendation | 6 | 6 |
| **Total** | **681** | **57** |
| | | |
| | Duplicates removed n=8 | **49 proceed to full-text screening** |

**S Table 4:**

Summary of articles from the searches or expert recommendation which passed abstract screening but which were excluded at full-text screening.

| Author | Year | DOI | Exclusion reason |
|---|---|---|---|
| Agirrezabal et al. | 2015 | 10.1016/j.jval.2015.03.258 | Published in abstract-only form |
| Alleweldt et al. | 2017 | 10.20506/rst.36.1.2631 | Review |
| Almomani et al. | 2020 | 10.1371/journal.pone.0238467 | Human DNA |
| Alterovitz et al. | 2018 | 10.1371/journal.pbio.3000099 | No economic analysis |
| Arthofer et al. | 2011 | 10.1007/s00438-011-0641-0 | No economic analysis |
| Bezdicek et al. | 2021 | 10.1038/s41598-021-96148-3 | No economic analysis |
| Buchanan-Hughes et al. | 2015 | 10.1016/j.jval.2015.09.1470 | Published in abstract-only |
| Buchanan and Wordsworth | 2019 | 10.1007/s41669-018-0101-4 | Review, human DNA |
| Ceyssens et al. | 2016 | 10.1093/jac/dkw201 | No economic analysis |
| Chai et al. | 2018 | 10.1371/journal.pone.0194648 | Did not consider WGS surveillance |
| Christensen et al. | 2018 | 10.3390/jpm5040470 | Human DNA |
| Deverka and Haga | 2015 | 10.1373/clinchem.2014.223412 | Human DNA |
| Dias-Neto et al. | 2009 | 10.1371/journal.pone.0008338 | Phage DNA |
| Graves, Garbett, Zhou and Peterson | 2017 | National Bureau of Economic Research Working Paper http://www.nber.org.liverpool.idm.oclc.org/papers/w24134.pdf | Human DNA |
| Gray | 2015 | 10.1016/j.jhin.2014.11.004 | Review |
| Hassan et al. | 2020 | 10.3390/microorganisms8111636 | No economic analysis |
| Hayden | 2020 | 10.1093/cid/ciz667 | Review |
| Hedman, Vasco and Zhang | 2020 | 10.3390/ani10081264 | No economic analysis |
| Kaprou, Bergšpica, Alexa, Alvarez-Ordóñez and Prieto | 2021 | 10.3390/antibiotics10020209 | Review |
| Lee, Izumiya, Iyoda and Ohnishi | 2019 | 10.1128/AEM.00728-19 | No economic analysis |
| Mitropoulos et al. | 2015 | 10.1186/s40246-015-0033-3 | Review, human DNA |
| Muellner, Stärk, Dufour, and Zadoks | 2016 | 10.1111/zph.12230 | Review |
| Muthuirulandi Sethuvel et al. | 2019 | 10.4103/ijmm.IJMM_19_396 | Review, no economic analysis |
| Oladeinde et al. | 2021 | 10.1128/mSystems.00729-21 | No economic analysis |

| | | | |
|---|---|---|---|
| Oliver et al. | 2014 | 10.1016/j.envint.2013.12.016 | No economic analysis, did not evaluate WGS |
| Peacock et al | 2018 | 10.1099/mgen.0.000173 | Did not evaluate WGS as a pathogen surveillance tool |
| Philips et al. | 2018 | 10.1016/j.jval.2018.06.017 | Review, human DNA |
| Plothner, Frank, von der Schulenburg and Matthias | 2017 | 10.1007/s10198-016-0815-0 | Human DNA |
| Regier, Weymann, Buchanan, Marshall and Wordsworth | 2018 | 10.1016/j.jval.2018.06.010 | Human DNA |
| Reuter et al. | 2013 | 10.1136/bmjopen-2012-002175 | No economic analysis |
| Roh, Abell, Kim, Nam and Bae | 2010 | 10.1016/j.tibtech.2010.03.001 | No economic analysis |
| Rosini, Nicchi, Pizza and Rappuoli | 2020 | 10.3389/fimmu.2020.01048 | Review, no economic analysis |
| Schürch and van Schaik | 2017 | 10.1111/nyas.13310 | Review, no economic analysis |
| Sekse et al. | 2017 | 10.3389/fmicb.2017.02029 | Review, no economic analysis |
| Stein, Martinez, Stiles, Miller and Zakharov | 2014 | 10.1371/journal.pone.0095525 | WGS not evaluated |
| Stratton, Schutzbank and Tang | 2021 | 10.1016/j.jmoldx.2021.09.003 | Review |
| Vozikis et al. | 2017 | 10.1159/000449152 | Review, human DNA |
| Weymann, Dragojlovic, Pollard and Regier | 2019 | 10.1007/s12687-019-00428-5 | Review, human DNA |
| Wordsworth et al. | 2018 | 10.1016/j.jval.2018.06.016 | Review |
| Zingg et al. | 2019 | 10.1186/s13756-019-0538-y | Review |

**S Table 5:**

Completed PRISMA checklist

| Section and Topic | Item # | Checklist item | Location where item is reported |
|---|---|---|---|
| **TITLE** | | | |
| Title | 1 | Identify the report as a systematic review. | Title page: “A systematic review of economic evaluations of whole genome sequencing for the surveillance of bacterial pathogens |
| **ABSTRACT** | | | |
| Abstract | 2 | See the PRISMA 2020 for Abstracts checklist. | S Table 5 |
| **INTRODUCTION** | | | |
| Rationale | 3 | Describe the rationale for the review in the context of existing knowledge. | Page 4: “WGS… can identify transmission links, describe outbreaks.. exclude outbreaks, and provide an understanding of antibiotic resistance in exquisite detail. While such information is unquestionably scientifically valuable, its widescale deployment in clinical diagnostics or for national and international surveillance systems has been constrained in part by cost.. The COVID-19 pandemic necessitated massive upscaling in laboratory and bioinformatic expertise and capacity, embedding WGS surveillance into routine practice, demonstrating both the utility and feasibility of large-scale WGS, and highlighting the potential for application to other pathogens. The antimicrobial-resistance (AMR) crisis is an ever-growing global health concern, with an estimated 4.95 million associated deaths in 2019. However, the economic realities of large-scale surveillance for AMR remain poorly explored. |
| Objectives | 4 | Provide an explicit statement of the objective(s) or question(s) the review addresses. | Page 5: “This review aims to comprehensively summarise and review available evidence relating to the economic implications of the use of WGS in the surveillance of bacterial pathogens, following a systematic methodology and reporting framework. Of particular interest was the potential application to antimicrobial-resistant (AMR) pathogens.” |
| **METHODS** | | | |
| Eligibility criteria | 5 | Specify the inclusion and exclusion criteria for the review and how studies were grouped for the syntheses. | Page 5-6: “Inclusion criteria were: published manuscripts or pre-print literature in English, available in full text between 1/10/1991 and 1/10/2021 with any form of full or partial economic evaluation of WGS for surveillance of one or more bacterial genus and/or species of World Health Organization (WHO)-defined priority pathogens for research and development of new antibiotics. Studies were included whether or not the threshold of drug-resistance was met… Duplicate studies, those which did not report an economic analysis, or which did not include surveillance for at least one of the priority pathogen species were excluded, due to our focus on antimicrobial resistance). Reviews and other forms of literature not representing primary analyses were not included in the review, although these were considered for background context. |

| Section and Topic | Item # | Checklist item | Location where item is reported |
|---|---|---|---|
| Information sources | 6 | Specify all databases, registers, websites, organisations, reference lists and other sources searched or consulted to identify studies. Specify the date when each source was last searched or consulted. | Page 6: six databases (Pubmed, Scopus, EconLit, Cochrane Library, NHS Economic Evaluation Database (NHSEED) and BioRxiv/MedRxiv) were searched on November 8th 2021. Reference lists and articles suggested by experts in the field were also screened for inclusion. |
| Search strategy | 7 | Present the full search strategies for all databases, registers and websites, including any filters and limits used. | Supplementary materials: S Table 1 |
| Selection process | 8 | Specify the methods used to decide whether a study met the inclusion criteria of the review, including how many reviewers screened each record and each report retrieved, whether they worked independently, and if applicable, details of automation tools used in the process. | Page 6: "Titles and/or abstracts were screened against inclusion criteria by one reviewer (VP). Articles selected for full-text review were exported to Rayyan (14) and were assessed against inclusion criteria inclusion by two reviewers working independently (VP and LN), with disagreements resolved through discussion." |
| Data collection process | 9 | Specify the methods used to collect data from reports, including how many reviewers collected data from each report, whether they worked independently, any processes for obtaining or confirming data from study investigators, and if applicable, details of automation tools used in the process. | Page 6: "Data were extracted from each included study by one reviewer (VP) into an Microsoft Excel spreadsheet (Redmond, Washington, United States), and checked by another (LN)." |
| Data items | 10a | List and define all outcomes for which data were sought. Specify whether all results that were compatible with each outcome domain in each study were sought (e.g. for all measures, time points, analyses), and if not, the methods used to decide which results to collect. | Page 6: "outcome data were extracted: estimated or actual impact of WGS on burden of illness (accepting any study definition of burden of illness i.e. cases or deaths averted); the costs and cost savings of WGS programmes; and the results of any break-even analysis." |
| | 10b | List and define all other variables for which data were sought (e.g. participant and intervention characteristics, funding sources). Describe any assumptions made about any missing or unclear information. | Page 6: "Study characteristics (publication year, year of data collection, economic analysis type, country setting, viewpoint, target organism(s), surveillance application, reporting currency, comparator, WGS post per isolate, comparator cost per isolate), methodological details" |
| Study risk of bias assessment | 11 | Specify the methods used to assess risk of bias in the included studies, including details of the tool(s) used, how many reviewers assessed each study and whether they worked independently, and if applicable, details of automation tools used in the process. | Page 7: "The Drummond-Jefferson checklist, developed to improve the clarity of reporting for economic analyses of healthcare interventions (15), was used as an objective measure of quality. The checklist was completed for each study by two reviewers (VP, LN) independently, with disagreements resolved by consensus." |
| Effect measures | 12 | Specify for each outcome the effect measure(s) (e.g. risk ratio, mean difference) used in the synthesis or presentation of results. | Page 6: "burden of illness (accepting any study definition of burden of illness i.e. cases or deaths averted"<br>Page 7: Costs and cost savings are reported in 2020 United States Dollars to enable comparisons |
| Synthesis methods | 13a | Describe the processes used to decide which studies were eligible for each synthesis (e.g. tabulating the study intervention characteristics and comparing against the planned groups for each synthesis (item #5)). | Page 7: "The heterogeneity of economic analysis types, geopolitical contexts, surveillance scales, timepoints and limited number of manuscripts precluded formal meta-analysis, so a narrative approach was taken to the synthesis of the methodology and results of included studies following the recommendations of the Synthesis |

| Section and Topic | Item # | Checklist item | Location where item is reported |
|---|---|---|---|
| | | | Without Meta-analysis (SWiM) reporting guideline” |
| | 13b | Describe any methods required to prepare the data for presentation or synthesis, such as handling of missing summary statistics, or data conversions. | N/A |
| | 13c | Describe any methods used to tabulate or visually display results of individual studies and syntheses. | Tables 2-3 |
| | 13d | Describe any methods used to synthesize results and provide a rationale for the choice(s). If meta-analysis was performed, describe the model(s), method(s) to identify the presence and extent of statistical heterogeneity, and software package(s) used. | Page 7: “In lieu of meta-analysis, a vote count on direction of effect is included. Studies were judged to favour WGS over the comparator where a) benefits outweighed costs in a cost-benefit analysis, b) dominance was established in cost-effectiveness analysis, c) author judgement of realistic case-numbers averted in break-even analysis” |
| | 13e | Describe any methods used to explore possible causes of heterogeneity among study results (e.g. subgroup analysis, meta-regression). | Page 7: “Heterogeneity is explored through presentation of a method and results table comparing the differing approach of different studies and the diversity in reporting outcomes.” |
| | 13f | Describe any sensitivity analyses conducted to assess robustness of the synthesized results. | N/A |
| Reporting bias assessment | 14 | Describe any methods used to assess risk of bias due to missing results in a synthesis (arising from reporting biases). | N/A |
| Certainty assessment | 15 | Describe any methods used to assess certainty (or confidence) in the body of evidence for an outcome. | N/A |
| **RESULTS** | | | |
| Study selection | 16a | Describe the results of the search and selection process, from the number of records identified in the search to the number of studies included in the review, ideally using a flow diagram. | Figure 1, S Table 2<br>Page 8: “662 studies were generated by the search strategy. Following title and abstract screening, 54 were identified for full-text assessment, of which 8 were duplicates and 46 proceeded to full-text screening. Screening of reference lists from included articles yielded one further article meeting the inclusion criteria. Nine studies were selected for inclusion in this review” |
| | 16b | Cite studies that might appear to meet the inclusion criteria, but which were excluded, and explain why they were excluded. | S Table 3 |
| Study characteristics | 17 | Cite each included study and present its characteristics. | Table 1 |
| Risk of bias in studies | 18 | Present assessments of risk of bias for each included study. | Table 4 |
| Results of individual studies | 19 | For all outcomes, present, for each study: (a) summary statistics for each group (where appropriate) and (b) an effect estimate and its precision (e.g. confidence/credible interval), ideally using structured | Table 2 |

| Section and Topic | Item # | Checklist item | Location where item is reported |
|---|---|---|---|
| | | tables or plots. | |
| Results of syntheses | 20a | For each synthesis, briefly summarise the characteristics and risk of bias among contributing studies. | N/A |
| | 20b | Present results of all statistical syntheses conducted. If meta-analysis was done, present for each the summary estimate and its precision (e.g. confidence/credible interval) and measures of statistical heterogeneity. If comparing groups, describe the direction of the effect. | Page 19: "In view of the lack of a unifying outcome measure reported by the studies, a vote count on the direction of effect was used to enable comparison. All included studies favoured the use of WGS over comparators." |
| | 20c | Present results of all investigations of possible causes of heterogeneity among study results. | N/A |
| | 20d | Present results of all sensitivity analyses conducted to assess the robustness of the synthesized results. | N/A |
| Reporting biases | 21 | Present assessments of risk of bias due to missing results (arising from reporting biases) for each synthesis assessed. | N/A |
| Certainty of evidence | 22 | Present assessments of certainty (or confidence) in the body of evidence for each outcome assessed. | N/A |
| **DISCUSSION** | | | |
| Discussion | 23a | Provide a general interpretation of the results in the context of other evidence. | Page 23: "The available evidence for the potential economic benefit of whole genome sequencing of AMR pathogen surveillance is heterogenous and of varying completeness, but broadly suggests that WGS can be economically viable from the public health perspective of food borne illnesses, and at the smaller scale of hospital IPC. We found that costs for single WGS test ranged from $72.13 to $470.37. Over the short timescale of the included studies (2019-2021) there was no evidence of WGS cost falling over time. In addition, there was no apparent regional variation in WGS cost, though all studies were in high and upper middle income nations with good supply chains. These costs are broadly in keeping with the costs per isolate identified by Raven et al., who reported prices for commercial sequencing of MRSA ranging between GBP 155-342 per isolate" |
| | 23b | Discuss any limitations of the evidence included in the review. | Page 25: "The difference in study design and methodology also make comparisons of quality assessment challenging.. |
| | 23c | Discuss any limitations of the review processes used. | Page 25: "Inclusion and exclusion criteria were intentionally broad.. [resulting] in marked heterogeneity, limiting the ability to make direct comparisons or aggregate results, precluding meta-analysis… The decision to focus on pathogens within the WHO list of priority pathogens for research and development of new antibiotics means that some important pathogens for which WGS surveillance is utilised were excluded, notably Mycobacterium tuberculosis (TB)… While a decision to perform narrative synthesis was made a priori, the form of synthesis was determined post hoc, with |

| Section and Topic | Item # | Checklist item | Location where item is reported |
|---|---|---|---|
| | | | potential for resulting inadvertent bias. It is possible that economic analyses of WGS surveillance showing negative implications may be less likely to be published, and we were unable to assess for this potential publication bias in this review.” |
| | 23d | Discuss implications of the results for practice, policy, and future research. | Page 23: “Future economic analyses of WGS should increasingly be able to use effectiveness measures from actual WGS surveillance programmes, rather than relying on assumed or modelled values.”<br>Page 24: “Future studies assessing the impact of WGS on hospital IPC should use actual data on the effectiveness of WGS surveillance rather than using historic outbreak data derived by conventional means.”<br>“Future studies are needed focusing specifically on the application of WGS surveillance to AMR.”<br>“Almost all of the settings evaluated were high income, but the burden of both food-borne illness AND AMR are higher in low-and-middle income countries (LMIC), particularly in regions of Africa. Understanding the costs and benefits of WGS surveillance in LMIC settings could have important implications not only for the health of these populations, but also for global efforts to tackle infectious illness, and in particular AMR.” |
| **OTHER INFORMATION** | | | |
| Registration and protocol | 24a | Provide registration information for the review, including register name and registration number, or state that the review was not registered. | Page 5: “registration on PROSPERO (registration number CRD42021289030” |
| | 24b | Indicate where the review protocol can be accessed, or state that a protocol was not prepared. | Page 5: “registration on PROSPERO (registration number CRD42021289030” |
| | 24c | Describe and explain any amendments to information provided at registration or in the protocol. | N/A |
| Support | 25 | Describe sources of financial or non-financial support for the review, and the role of the funders or sponsors in the review. | Page 5: “There was no specific funding for this review.” |
| Competing interests | 26 | Declare any competing interests of review authors. | Competing interests section |
| Availability of data, code and other materials | 27 | Report which of the following are publicly available and where they can be found: template data collection forms; data extracted from included studies; data used for all analyses; analytic code; any other materials used in the review. | N/A |

**S Table 6:**

Completed PRISMA Abstract checklist

| Section and Topic | Item # | Checklist item | Reported (Yes/No) |
|---|---|---|---|
| **TITLE** | | | |
| Title | 1 | Identify the report as a systematic review. | Yes |
| **BACKGROUND** | | | |
| Objectives | 2 | Provide an explicit statement of the main objective(s) or question(s) the review addresses. | Yes |
| **METHODS** | | | |
| Eligibility criteria | 3 | Specify the inclusion and exclusion criteria for the review. | Yes |
| Information sources | 4 | Specify the information sources (e.g. databases, registers) used to identify studies and the date when each was last searched. | Yes |
| Risk of bias | 5 | Specify the methods used to assess risk of bias in the included studies. | Yes |
| Synthesis of results | 6 | Specify the methods used to present and synthesise results. | Yes |
| **RESULTS** | | | |
| Included studies | 7 | Give the total number of included studies and participants and summarise relevant characteristics of studies. | Yes |
| Synthesis of results | 8 | Present results for main outcomes, preferably indicating the number of included studies and participants for each. If meta-analysis was done, report the summary estimate and confidence/credible interval. If comparing groups, indicate the direction of the effect (i.e. which group is favoured). | Yes |
| **DISCUSSION** | | | |
| Limitations of evidence | 9 | Provide a brief summary of the limitations of the evidence included in the review (e.g. study risk of bias, inconsistency and imprecision). | Yes |
| Interpretation | 10 | Provide a general interpretation of the results and important implications. | Yes |
| **OTHER** | | | |
| Funding | 11 | Specify the primary source of funding for the review. | Yes |
| Registration | 12 | Provide the register name and registration number. | Yes |

**S Table 7:**

Completed SWiM checklist

| **SWiM reporting item** | **Description** | **Page in manuscript** |
|---|---|---|
| **Methods** | | |
| 1 Grouping studies for synthesis | 1a) Provide a description of, and rationale for, the groups used in the synthesis (e.g., groupings of populations, interventions, outcomes, study design) | 7 |
| | 1b) Detail and provide rationale for any changes made subsequent to the protocol in the groups used in the synthesis | 7 |
| 2 Describe the standardised metric and transformation methods used | Describe the standardised metric for each outcome. Explain why the metric(s) was chosen and describe any methods used to transform the intervention effects, as reported in the study, to the standardised metric, citing any methodological guidance consulted | 7 |
| 3 Describe the synthesis methods | Describe and justify the methods used to synthesise the effects for each outcome when it was not possible to undertake a meta-analysis of effect estimates | 7 |
| 4 Criteria used to prioritise results for summary and synthesis | Where applicable, provide the criteria used, with supporting justification, to select the particular studies, or a particular study, for the main synthesis or to draw conclusions from the synthesis (e.g., based on study design, risk of bias assessments, directness in relation to the review question) | N/A |
| 5 Investigation of heterogeneity in reported effects | State the method(s) used to examine heterogeneity in reported effects when it was not possible to undertake a meta-analysis of effect estimates and its extensions to investigate heterogeneity | Table 3 |
| 6 Certainty of evidence | Describe the methods used to assess the certainty of the synthesis findings | N/A |
| 7 Data presentation methods | Describe the graphical and tabular methods used to present the effects (e.g., tables, forest plots, harvest plots) | Tables 2-3 |
| | Specify key study characteristics (e.g., study design, risk of bias) used to order the studies, in the text and any tables or graphs, clearly referencing the studies included | Table 4 |
| **Results** | | |
| 8 Reporting results | For each comparison and outcome, provide a description of the synthesised findings and the certainty of the findings. Describe the result in language that is consistent with the question the synthesis addresses, and indicate which studies contribute to the synthesis | Table 2 and page 21 |
| **Discussion** | | |
| 9 Limitations of the synthesis | Report the limitations of the synthesis methods used and/or the groupings used in the synthesis and how these affect the conclusions that can be drawn in relation to the original review question | 24 |
